# Low clinical impact of HIV drug resistance mutations in oral pre-exposure prophylaxis: a systematic review and meta-analysis

**DOI:** 10.1186/s12981-024-00627-2

**Published:** 2024-06-06

**Authors:** Brian Eka Rachman, Siti Qamariyah Khairunisa, Citrawati Dyah Kencono Wungu, Tri Pudy Asmarawati, Musofa Rusli, Bramantono, M. Vitanata Arfijanto, Usman Hadi, Masanori Kameoka, Nasronudin

**Affiliations:** 1 Subspeciality Program in Internal Medicine, Faculty of Medicine, Airlangga University, Surabaya, Indonesia; 2 Institute of Tropical Disease, Airlangga University, Surabaya, Indonesia; 3 Department of Physiology and Medical Biochemistry, Faculty of Medicine, Airlangga University, Surabaya, Indonesia; 4 Division of Tropical and Infectious Diseases, Department of Internal Medicine, Faculty of Medicine, Airlangga University, Surabaya, Indonesia; 5 Department of Public Health, Kobe University Graduate School of Health Sciences, 7-10-2 Tomogaoka, Suma-Ku, Kobe, Hyogo 654-0142 Japan; 6 Center for Infectious Diseases, Kobe University Graduate School of Medicine, Hyogo, Japan

**Keywords:** Pre-exposure prophylaxis, Mutation, HIV, Drug resistance mutation, Tenofovir, Emtricitabine

## Abstract

**Introduction:**

Despite the widespread use of pre-exposure prophylaxis (PrEP) in preventing human immunodeficiency virus (HIV) transmission, scant information on HIV drug resistance mutations (DRMs) has been gathered over the past decade. This review aimed to estimate the pooled prevalence of pre-exposure prophylaxis and its two-way impact on DRM.

**Methods:**

We systematically reviewed studies on DRM in pre-exposure prophylaxis according to the Preferred Reporting Items for Systematic Reviews and Meta-Analysis 2020 guidelines. PubMed, Cochrane, and SAGE databases were searched for English-language primary studies published between January 2001 and December 2023. The initial search was conducted on 9 August 2021 and was updated through 31 December 2023 to ensure the inclusion of the most recent findings. The registration number for this protocol review was CRD42022356061.

**Results:**

A total of 26,367 participants and 562 seroconversion cases across 12 studies were included in this review. The pooled prevalence estimate for all mutations was 6.47% (95% Confidence Interval-CI 3.65–9.93), while Tenofovir Disoproxil Fumarate/Emtricitabine-associated drug resistance mutation prevalence was 1.52% (95% CI 0.23–3.60) in the pre-exposure prophylaxis arm after enrolment. A subgroup analysis, based on the study population, showed the prevalence in the heterosexual and men who have sex with men (MSM) groups was 5.53% (95% CI 2.55–9.40) and 7.47% (95% CI 3.80–12.11), respectively. Notably, there was no significant difference in the incidence of DRM between the pre-exposure prophylaxis and placebo groups (log-OR = 0.99, 95% CI −0.20 to 2.18, I2 = 0%; p = 0.10).

**Discussion:**

Given the constrained prevalence of DRM, the World Health Organization (WHO) advocates the extensive adoption of pre-exposure prophylaxis. Our study demonstrated no increased risk of DRM with pre-exposure prophylaxis (p > 0.05), which is consistent with these settings. These findings align with the previous meta-analysis, which reported a 3.14-fold higher risk in the pre-exposure prophylaxis group than the placebo group, although the observed difference did not reach statistical significance (p = 0.21).

**Conclusions:**

Despite the low prevalence of DRM, pre-exposure prophylaxis did not significantly increase the risk of DRM compared to placebo. However, long-term observation is required to determine further disadvantages of extensive pre-exposure prophylaxis use.

PROSPERO Number: CRD42022356061.

**Supplementary Information:**

The online version contains supplementary material available at 10.1186/s12981-024-00627-2.

## Introduction

The persistent global public health challenge posed by the human immunodeficiency virus (HIV) epidemic remains a concern despite substantial efforts to limit its spread. In 2022, the global population of individuals living with HIV was estimated to be around 37.5 million, with an additional 1.3 million people newly infected with the virus [1]. Considering this ongoing situation, Pre-exposure prophylaxis (PrEP) has been introduced as a preventative measure for those at high risk of HIV infection. PrEP involves a medication regimen for individuals who are currently HIV-negative but are at a high risk of contracting the virus.

The efficacy of oral Pre-exposure Prophylaxis (PrEP) in preventing HIV has been thoroughly established, demonstrating a dramatic reduction in HIV incidence. A study indicates that individuals who maintain high adherence to the regimen can see reductions in transmission rate by up to 93% [2]. This effectiveness is consistent across diverse populations, including men who have sex with men and serodiscordant couples, where meta-analyses have confirmed PrEp’s robustness, achieving transmission reduction as high as 75% with consistent adherence [3]. Despite its effectiveness, concerns have been raised regarding the potential emergence of HIV drug resistance mutation (DRM) among PrEP users. Theoretically, these DRMs could compromise the effectiveness of current first-line antiretroviral therapy (ART) regimens if an individual contracts HIV while on PrEP. However, recent studies, including the NADIA trial, reported that mutations associated with Tenofovir Disoproxil Fumarate/Emtricitabine (TDF/FTC), the drugs used in PrEP, may not significantly impact the effectiveness of newer first-line ART regimens. This is particularly true for those regimens incorporating Integrase Strand Transfer Inhibitors (INSTIs) such as dolutegravir [4]. However, it is essential to note that the implications of TDF/FTC-associated mutations remain unclear for individuals who are intolerant to dolutegravir. Therefore, further research is needed to fully understand the potential impact of these mutations.

The World Health Organization recommends oral PrEP for individuals at substantial risk of HIV infection. In alignment with this, the U.S. Food and Drug Administration (FDA) has sanctioned two specific oral therapies. The first approval, granted in 2012, was for Emtricitabine-Tenofovir Disoproxil Fumarate (FTC/TDF). This was followed by the approval of Emtricitabine-Tenofovir Alafenamide (FTC/TAF) in 2019 [5]. In 2021, the recommendation was expanded to include the Dapivirine ring for women at substantial risk. By 2022, long-acting injectable Cabotegravir (CAB-LA) was also recommended for individuals at substantial risk of HIV. Recent advances in HIV PrEP, including the approval of CAB-LA, the continued use of oral PrEP therapies, and the exploration of new delivery methods, aim to improve adherence to PrEP and reach populations who may not have been reached by current forms of PrEP [6]. However, the focus of this study will be exclusively on oral PrEP, given its widespread application and the extensive research supporting its use.

The mechanism underlying drug resistance mutation to TDF and FTC involved multifaceted processes across biochemical, structural biology, genotypic, and phenotypic dimensions. Biochemically, mutations disrupt the binding affinity of the HIV reverse transcriptase enzyme, diminishing the inhibitory potency of the drugs [7]. Structural biology insights highlight atomic-level interaction, such as conformational changes induced by mutation like M184V, which reduce drug binding efficacy [8, 9]. Genotypically, mutations such as K65R and K70E interfere with drug-virus interactions, driven by the error-prone nature of HIV reverse transcriptase and drug exposure [10, 11]. Additionally, the relationship between adherence to pre-exposure prophylaxis (PrEP) regimens and efficacy significantly impacts the selection of drug resistance mutation. Suboptimal adherence can compromise PrEP efficacy, leading to an increased risk of breakthrough infections and selection pressure for drug-resistant variants [12]. This understanding underscores the complexity of the resistance mechanism and emphasizes the importance of comprehensive approaches in combatting HIV drug resistance mutation while ensuring optimal adherence to PrEP regimens.

The relationship between adherence to PrEP, its efficacy, and its influence on DRM selection has been the subject of extensive research. However, clinical trials have reported inconsistent findings regarding the presence of DRMs in individuals who contract HIV while on PrEP [13–15]. A previous non-systematic review of randomized controlled trials (RCTs) on PrEP indicated a DRM prevalence of 5.9% [3, 16], highlighting the necessity for a more comprehensive evaluation. It is important to note that the primary focus of previous meta-analyses on oral PrEP has been efficacy and safety outcomes [3, 17]. This points to the need for more focused research on the aspect of DRM in the context of PrEP usage.

Given the concerns surrounding HIV drug resistance mutations, particularly transmitted HIV drug resistance mutations, this study aims to assess its prevalence and clinical implications for individuals who experience seroconversion while using oral Pre-Exposure Prophylaxis (PrEP). The findings could serve as a basis for future research into the impact of these mutations on HIV treatment options after PrEP failure.

## Methods

### Study designs

The systematic literature review followed the guidelines from the Preferred Reporting Items for Systematic Reviews and Meta-Analyses (PRISMA) [18]. The study protocol was registered in the International Prospective Register of Systematic Reviews (PROSPERO) (https://www.crd.york.ac.uk/prospero/) under registration number CRD42022356061.

### Search strategy

Relevant data were obtained via the PubMed, SAGE, and Cochrane databases for studies published between January 2001 and December 2020. The initial search was conducted on 9 August 2021, and subsequently, the search was updated through 31 December 2023 to ensure the inclusion of the most recent findings in this paper. Reference citations were manually explored to optimize the findings and reduce publication bias. The search procedure for each database is shown in Table S1 Table in the (S1 File).

### Study selection

The records obtained from the search process were imported into Rayyan, a web application for systematic reviews with blind coding features [19]. The titles and abstracts were selected after eliminating duplicate articles. Subsequently, full texts of the articles were retrieved before assessing their conformity to the inclusion and exclusion criteria. Each investigator recorded the reasons for exclusion during the screening process using specific labels. Each selection step was performed blindly by two independent investigators (BER and SQK), who were then unblinded to show discrepancies between the two investigators. A third investigator (MK) served as a mediator during discussions to reach a consensus on the results.

### Eligibility criteria

Studies that met the following criteria were included in the systematic review: (1) used a clinical trial design; (2) were in the English language; (3) provided HIV-1 mutation test results and/or PrEP-related DRMs; and (4) enrolled participants that were over 18 years of age. We excluded studies based on the following criteria: (1) scientific work in the form of case reports, poster reports, correspondences, commentary, and review articles; (2) not available in full text; (3) duplicate studies; and (4) irrelevant outcomes.

### Quality assessment

Methodological quality assessment was conducted by two investigators (BER and SQK) to evaluate the risk of bias in eligible studies using the Cochrane Collaboration's Risk of Bias 2 (RoB 2) tool [20]. Any discrepancies between the two investigators were resolved through dialogue mediated by a third investigator (MK). The RoB 2 tool assessed five domains that could bias clinical trial study: the randomization process, bias risk due to deviations from the intended interventions (effect of assignment to intervention effect), bias risk due to adhering to intervention, missing outcome data, bias risk in outcome measurement, and bias risk in result selection. Each domain was graded as low, high, or some concern and the overall bias risk was assessed.

### Data extraction

Two investigators (BER and SQK) independently extracted data from each study and engaged in collaborative discussions to resolve conflicts in study selection. In disagreements, a third investigator (MK) made the definitive determination. The author's name, publication year, study location, sample size, study population, examination tool, PrEP regimen, and mutation type were retrieved from eligible papers (Table 1). All mutations and the prevalence of TDF/FTC-associated DRMs in each study were outcomes documented from data retrieval (S2 Table). The authors were contacted to obtain additional information when incomplete data were identified during the extraction process. All HIV-1 mutations found in the PrEP clinical trial studies were defined as all mutations, regardless of the antiretroviral phenotype. TDF/FTC-associated DRMs were described as mutations that induce TDF resistance, such as K65R and K70E. Otherwise, they were defined as mutations that caused FTC resistance, such as M184V/I/M. Mutations at enrolment were defined as mutations discovered during enrolment screening or seroconversion within four weeks of enrolment. Meanwhile, mutations after enrolment were defined as those found more than four weeks after randomization.

**Table 1:** Characteristics of the included studies on HIV drug resistance mutations among people initiating pre-exposure prophylaxis

No	First Author	Year	PrEP^1^ regimen	Sample size (Seroconversion)	Country	Study population	Study design	Molecular technique used for genotyping	Mutation variant	ROB2^2^
1	Thigpen et al. (TDF2) [35]	2012	TDF^3^-FTC^4^	26	Botswana (Africa)	Heterosexual	Arm1: TDF-FTC (oral) Arm2: placebo (oral)	Genotype: Qiagen BioRobot M48	Major	Low
2	Liegler et al. (iPrex) [25, 40]	2014	TDF-FTC	141	South Africa, Brazil, Thailand, and the United States (Africa, America, Asia)	MSM^5^	Arm1: TDF-FTC (oral) Arm2: placebo (oral)	Genotype: TRUGENE, Siemens qMVA (minor variant)	Major	Low
3	Grant et al. (FEM) [26, 41]	2015	TDF-FTC	68	Kenya, Tanzania (Africa)	Heterosexual	Arm1: TDF-FTC (oral) Arm2: placebo (oral)	Genotype: TRUGENE, Siemens Phenotype: PhenoSense, monogram biosciences AS-PCR^6^ + 454 platform (minor variant)	Major, Minor	Low
4	Marrazzo et al. (VOICE) [13]	2015	TDF, TDF-FTC, Placebo 1% TFV^7^ vaginal gel	140	Uganda, Zimbabwe (Africa)	Heterosexual	Arm1: TDF (oral) Arm2: TDF-FTC (oral) Arm3: placebo (oral) Arm4: 1% TFV (gel) Arm5: placebo (gel)	Genotype: ViroSeq	Major	Low
5	Zhang et al. (HPTN 073) [15]	2017	TDF-FTC	8	United States (America)	MSM	Non-placebo-controlled study	Genotype: ViroSeq & NGS^8^	Major, Minor	High
6	Sivay et al. (ADAPT) [30]	2017	TDF-FTC	12	South Africa, Thailand, United States (Africa, America, Asia)	Heterosexual, MSM	Non-placebo-controlled study (daily usage/time-driven usage/event-driven usage)	Genotype: ViroSeq & NGS	Major	Low
7	Delaugerre et al. (IPERGAY) [24, 54]	2018	TDF-FTC	31	France, Canada (Europe)	MSM	Arm1: TDF-FTC (oral) Arm2: placebo (oral) (BLIND^9^/OLE^10^ phase)	Sanger + UDS^11^ (454 platform)	Major, Minor	Low
8	Lehman et al. [32, 33, 52],	2015	TDF, TDF-FTC	83	Kenya, Uganda (Africa)	Heterosexual	Arm1: TDF (oral) Arm2: TDF-FTC (oral) Arm3: placebo (oral)	Genotype: ViroSeq	Major, Minor	Low
9	McCormack et al. (PROUD) [14]	2016	TDF-FTC	26	England (Europe)	MSM	Non-placebo-controlled study (Immediate group/Deferred group)	NA^12^	Major	Low
10	Liu et al. (DEMO) [31]	2016	TDF-FTC	5	United States (America)	MSM	Non-placebo-controlled study	Genotype: TRUGENE, Siemens qMVA (minor variant)	Major	High
11	Mayer et al. (DISCOVER) [33]	2020	TDF-FTC, TAF^13^-FTC	15	Austria, Denmark, France, Germany, Ireland, Italy, Netherlands, Spain, UK, Canada, United States (Europe, America)	MSM	Non-placebo-controlled study Arm1: TDF-FTC (oral) Arm2: TAF-FTC (oral)	NA	Major	Low
12	Phanuphak et al. (Princess) [32]	2018	TDF-FTC	7	Thailand (Asia)	MSM	Non-placebo-controlled study	NA	Major	High

^1^*PrEP* Pre-Exposure Prophylaxis

^2^*RoB 2* Risk of Bias 2

^3^*TDF* Tenofovir Disoproxil Fumarate

^4^*FTC* Emtricitabine

^5^*MSM* Men who have sex with men

^6^*PCR* Polymerase Chain Reaction

^7^*TFV* Tenofovir

^8^*NGS* Next-Generation Sequencing

^9^*BLIND* The double-blind randomized phase

^10^*OLE* The open-label phase

^11^*UDS* Ultradeep Sequencing

^12^*NA* Not available

^13^*TAF* Tenofovir alafenamide

### Statistical analysis

#### Main analysis

All analyses were performed using STATA software (version 17.0; Stata Corporation, College Station, TX, USA). The pooled prevalence was analyzed and calculated with a 95% confidence interval (CI) for all mutations that occurred in the PrEP clinical trial studies. This analysis was also extended to the pooled prevalence of the following mutations: resistance mutations associated with TDF and/or FTC resistance at enrolment and mutations associated with TDF and/or FTC resistance in the total PrEP arm after enrolment, Metaprop in Stata was used to supplement the methane command to perform a pooled prevalence meta-analysis [21].

This study also analyzed the differences in DRM rates associated with TDF and/or FTC resistance in the arm receiving the PrEP regimen compared with the placebo arm. For this purpose, the approach proposed by Bradburn et al. was used for the meta-analysis of rare events [22]. If event rates are less than 1%, there is no significant size disparity between the treatment and control groups in clinical trial studies, the treatment effect is not disproportionately significant, and the Peto one-step odds ratio method is the most appropriate and least biased method. However, if these conditions are not met, the Mantel–Haenszel odds ratio analysis method without zero-cell corrections may be used. Cochran's Q statistic was used to analyze study heterogeneity, and Higgins’ I^2^ statistic was used to quantify it. The I^2^ values of 0%, 25%, 50%, and 75% indicated no, low, moderate, and high heterogeneity, respectively. A p-value for heterogeneity was used to determine whether to use a fixed- or random-effects model. The random-effects model was used if the p-value was less than 0.05; otherwise, the fixed-effects model was used.

#### Publication bias, sensitivity, and subgroup analysis

To identify the effects of limited sample sizes and other potential reporting biases, publication bias was quantitatively evaluated using Egger's regression test and assessed visually using the funnel plot [23]. The leave-one-out method was used to perform the sensitivity analysis, excluding each study from the one-at-a-time analysis. Subgroup analysis was conducted to measure the variation in effect size across subgroups and the influence of particular covariates on aggregated outcomes. Subgroup analysis will be performed according to (1) study locations and (2) study population.

## Results

### Study selection overview

**Fig. 1:**
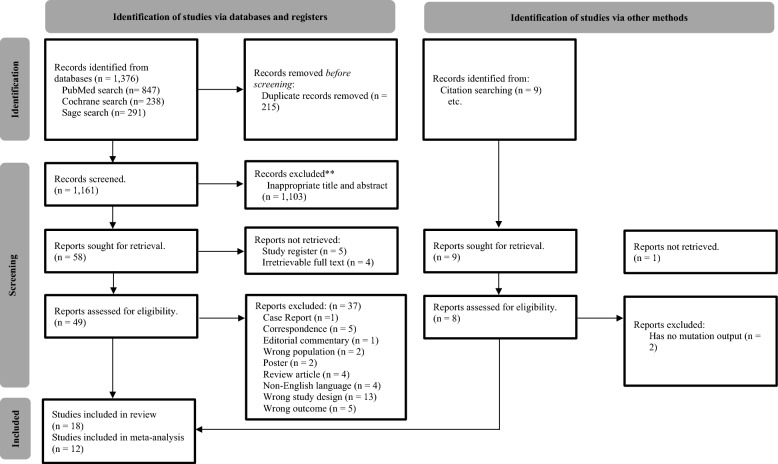
PRISMA flow diagram of the study selection process

A total of 1,376 studies matched the keywords in three databases, PubMed, Cochrane, and SAGE, following the PRISMA flowchart guidelines, as schematically represented in (Fig. 1). We excluded 215 duplicate studies and 1103 studies whose titles and abstracts did not meet the inclusion criteria. Nine of the 58 studies were excluded because five were study registries, and full-text access was unavailable for the other four studies. In addition to conducting a database search, nine studies were identified through citation searches. However, the full text of one study was unavailable. Subsequently, the eligibility of 57 full-text studies was evaluated, and 39 were considered ineligible because they comprised case reports, correspondences, editorial commentaries, posters, and review articles, were not in English, were not clinical trials, and did not have relevant outcomes. In total, 18 studies met the eligibility criteria for inclusion in the systematic review. However, only 12 of these studies were quantitatively analyzed, as some articles originated from the same study [24–29].

### Characteristics and outcomes of the included studies

The characteristics of the included studies are summarized in Table 1. There were 26,367 adult participants involved in 12 studies. Among these, six studies compared the oral TDF-FTC PrEP use with an oral placebo. One study compared the oral TDF-FTC PrEP with a 1% Tenofovir (TFV) and placebo gel. Another study compared the oral TDF-FTC PrEP with Tenofovir Alafenamide-Emtricitabine (TAF-FTC) oral PrEP. One study compared the outcomes between daily usage, time-driven usage, and event-driven usage of PrEP. Finally, one study compared the results between an immediate group that started the PrEP regiment immediately and a deferred group that started the regiment later. In addition, three non-placebo-controlled studies provided valuable data for meta-analysis. Most of the studies were conducted in Africa (n = 4), followed by Europe (n = 2), the United States (n = 2), and Asia (n = 1). Three additional studies were conducted simultaneously in Africa, Asia, and the United States. The MSM group comprised the most eligible studies (n = 7), followed by the heterosexual population (n = 4); the remaining included both populations. Based on the mutation detection instrument, four studies used ViroSeq genotype examination, three used TruGene, and one used Qiagen and Sanger. Several studies have utilized NGS (n = 2) for precision analyses. All the included studies reported significant phenotypic mutations (n = 8), although some also reported minor variants (n = 4) (Table 1). The characteristics of all drug-resistance mutations are summarized in (S2 Table). In the studies analyzed, a total of 48 mutations were identified. Among all DRMs associated with the PrEP TDF-FTC regimen, FTC resistance mutations were the most prevalent. Specifically, M184V was found in 16 of the 48 mutations (33.3%), followed by M184I in 11 of the 48 mutations (22.9%). The mutations M184IV, M184MV, M184MIV, and M184MI were found in 8 (16.6%), 3 (6.2%), 2 (4.1%), and 1 (2%) of the 48 mutations, respectively. The TDF DRM K65R was present in 7 of the 48 mutations (14.5%) identified across the trial participants. K70E was not detected in the present study.

### Study quality assessment

Study quality was assessed using a domain-specific quality assessment with Cochrane's RoB 2 tool. The results are shown in (S1(A) Fig), and a detailed evaluation of the risk of bias in each study is shown in (S1(B) Fig). Three of the twelve studies evaluated exhibited a high risk of overall bias. Due to insufficient information, the first study had a high risk of bias in the randomization process domain. Some studies were found to possess a high risk of bias within the domain of the randomization process, attributed to inadequate information and study design [14, 15, 30–32]. Some concerns were also raised regarding deviations from the intended intervention domain because of a need for more information regarding the blinding technique [15]. Furthermore, a particular study exhibited some notable concerns in the domain of randomization procedures due to insufficient information regarding the concealment of allocation sequences until participants were enrolled and assigned to interventions [33].

### Primary outcomes

**Fig. 2:**
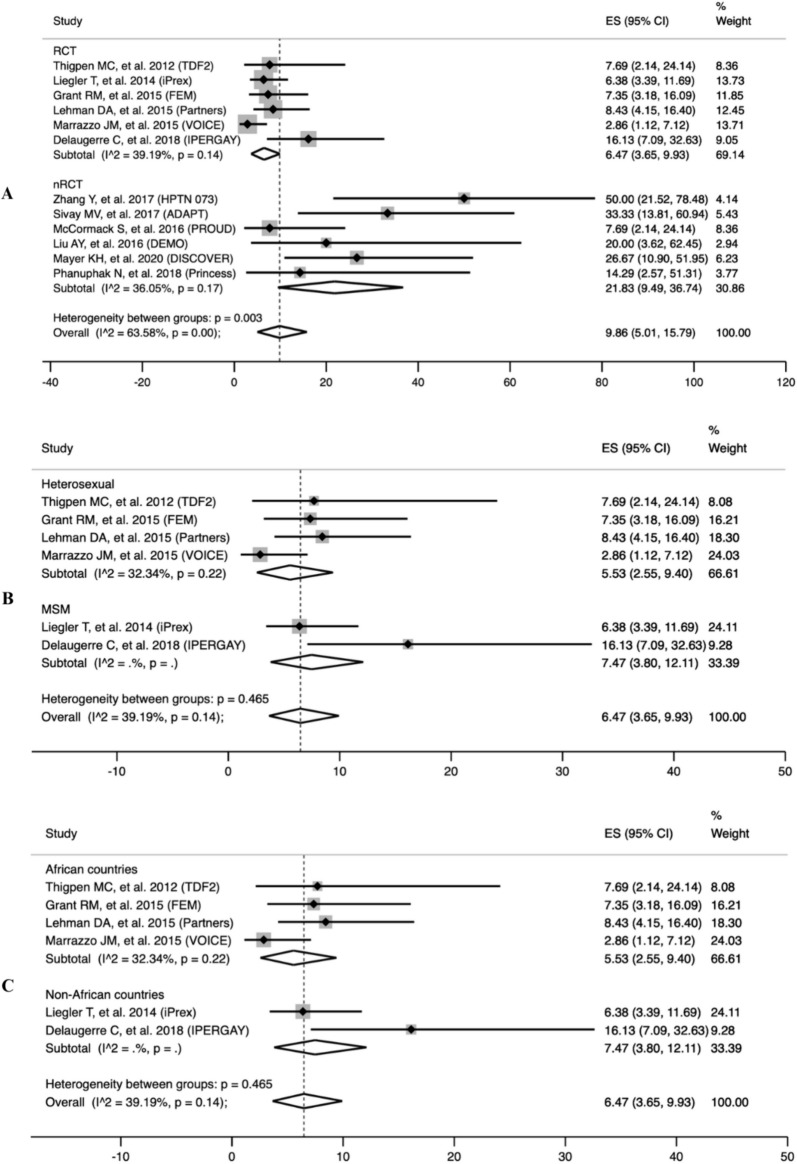
The pooled prevalence of all mutations. **A** Subgroup analysis based on study design **B** Subgroup analysis based on study population in RCT studies. **C** Subgroup analysis based on the study location in RCT studies. *ES* Effect Size is equivalent to prevalence, *CI* confidence interval

**Fig. 3:**
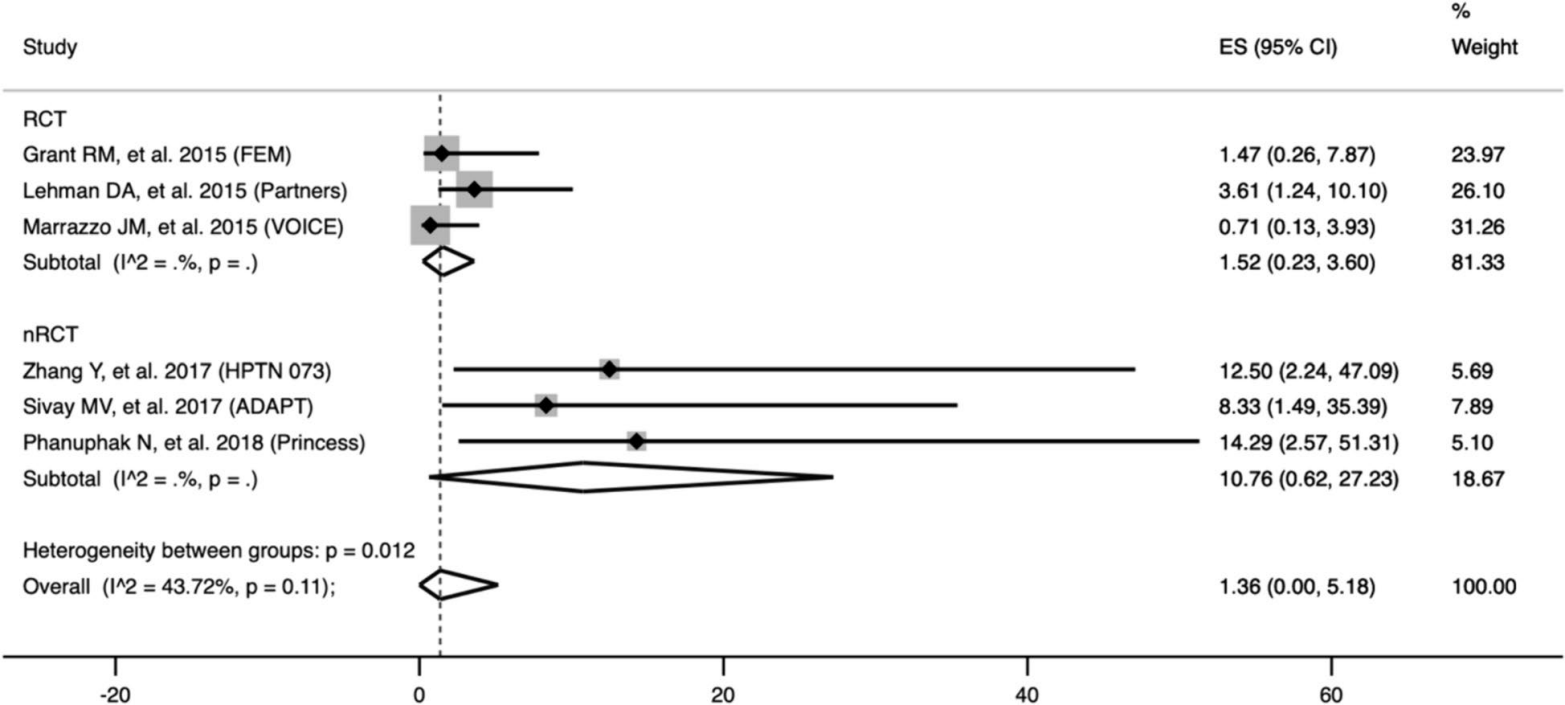
The pooled prevalence of TDF/FTC-associated drug resistance mutation in oral pre-exposure prophylaxis arm after enrolment. *ES* Effect size equivalent to prevalence, *CI* confidence interval

**Fig. 4:**
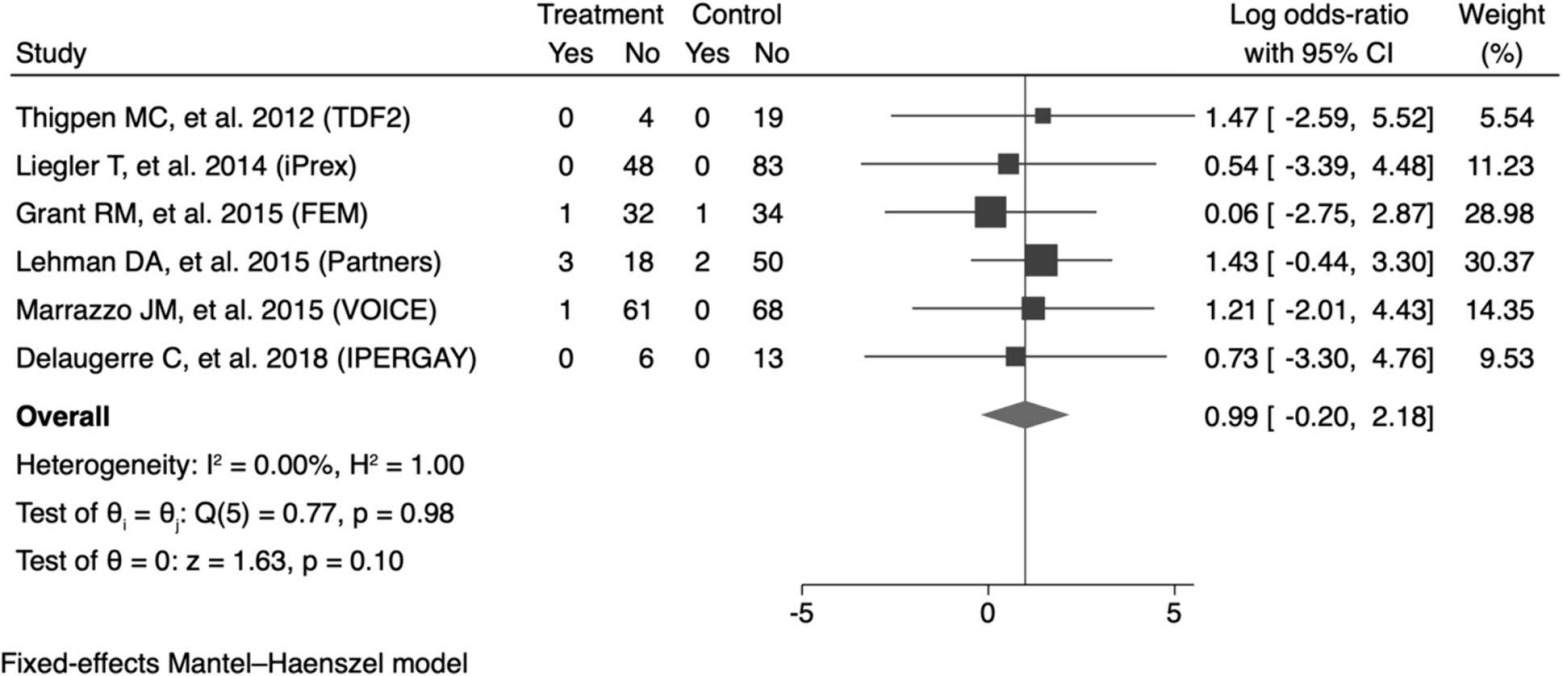
The risk of TDF/FTC-associated HIV drug resistance mutation in oral pre-exposure prophylaxis arm after enrolment. *CI* confidence interval

In total, 562 seroconversion cases during pre-exposure prophylaxis were observed to measure the pooled prevalence of HIV drug-resistant mutations. We found that the pooled prevalence of all seroconversion mutations was 9.86% (95% CI 5.01–15.79), with high heterogeneity among the studies (I^2^ = 63.58%; p = 0.00). Furthermore, the DRM pooled prevalence in randomized controlled trial studies (RCTs) was found to be 6.47% (95% CI 3.65–9.93). For non-randomized controlled trial studies (nRCTs), the DRM pooled prevalence was significantly higher at 21.83% (95% CI 9.49–36.74) (Fig. 2). At enrolment, the pooled prevalence of TDF/FTC-associated DRM was 4.07% (95% CI 1.12–8.2), with a high degree of heterogeneity among the studies (I^2^ = 56.05%; p = 0.02). Subsequently, the pooled prevalence, based on study design, was determined to be 2.48% (95% CI 1.09–4.28) in randomized controlled trial studies (RCTs), whereas, in non-randomized controlled trial studies (nRCTs), it exhibited a notably higher rate at 13.79% (95% CI 5.02–25.08) (S2 Fig). Within the after enrolment group, the pooled prevalence in the oral PrEP arm was 1.36% (95% CI 0–5.18), demonstrating low heterogeneity among the studies (I^2^ = 0%; p = 0.16). Subsequent analysis based on the study design revealed that the pooled prevalence among randomized controlled trial studies (RCTs) was 1.52% (95% CI 0.23–3.60). Conversely, in non-randomized controlled trial studies (nRCTs), the pooled prevalence was significantly higher at 10.76% (95% CI 0.62–27.23) (Fig. 3). Compared to the placebo incidence, the likelihood of TDF/FTC-associated DRMs is 0.99 (95% CI −0.20 to 2.18) in the group that received PrEP (I^2^ = 0.00%; p = 0.10) (Fig. 4). The leave-one-out sensitivity analysis revealed that no study significantly affected the aggregate effect size (S3 Table).

### Subgroup analysis

Subgroup analysis based on the study population was performed to identify the cause of the high heterogeneity in the pooled prevalence of all mutations. We found that the pooled prevalence of DRMs in the heterosexual population was 5.53% (95% CI 2.55–9.40). Meanwhile, the MSM population’s pooled prevalence was 7.47% (95% CI 3.80–12.11) (Fig. 2B). A subgroup analysis was also carried out based on the research's location, where the pooled prevalence in non-African countries was 7.47% (95% CI 3.80–12.11). This was followed by African countries’ pooled prevalence of 5.53% (95% CI 2.55–9.40) (Fig. 2C).

### Publication bias

Using a funnel plot, we found no evidence of bias in the studies included in the analysis of the prevalence of DRMs after PrEP administration (S3–S6 Figs). We observed an asymmetric distribution in the funnel plot of studies that reported the prevalence of all mutations in patients with PrEP (S3 Fig). These results were confirmed by Egger's test, which indicated that they were not statistically significant (p = 0.13). Furthermore, Egger’s test for pooled prevalence of TDF/FTC-associated drug resistance mutations at enrolment, the pooled prevalence of TDF/FTC-associated drug resistance mutations in the pre-exposure prophylaxis arm after enrolment, and risk of TDF/FTC-associated drug resistance mutations in the pre-exposure prophylaxis arm after enrolment also yielded insignificant results (p > 0.05).

## Discussion

In the context of HIV prevention, pre-exposure prophylaxis (PrEP) has emerged as an effective method for mitigating transmission rates. Nonetheless, the emergence and dissemination of HIV drug resistance mutation (DRM) in individuals using PrEP raises critical concerns about its long-term effectiveness and highlights the need for a comprehensive assessment of drug resistance mutation pooled prevalence in this population. Despite concerns about the emergence and dissemination of DRM in individuals using PrEP, our study revealed that the pooled prevalence of HIV drug resistance mutation was lower (1.52%) than predicted by a prior mathematical model (2–4%) [34]. This discrepancy could be attributed to the inclusion of undiagnosed acute infections in the model’s calculation, as most PrEP HIV drug resistance mutations have been detected in this situation, significantly influencing the findings [26, 28, 35]. Specifically, our study examined DRM discovered in the group that received PrEP after enrolment and during screening separately. In contrast, the prevalence of DRM in our study was slightly higher than that reported in previous studies, which indicated a prevalence of 0.18% [16]. The disparities in the findings between our study and the previous one arise from distinct research designs. Unlike prior studies that encompassed all PrEp-exposed clinical trial participants, our study was exclusively focused on individuals who were seroconverted. Furthermore, our study took into account the weightage of each study in the pooled analysis, which provides a more nuanced understanding of the data, thereby enhancing the robustness of our findings. Besides that, as previously noted, the prevalence of DRM in PrEP clinical trials tends to be lower compared to observational studies and real-world settings [36]. This discrepancy can be attributed to the stringent adherence monitoring and participant selection protocols typically employed in clinical trials, which may constrain the applicability of their findings to broader populations. Therefore, it is imperative to investigate the impact of PrEP on DRM in real-world settings, characterized by potentially lower adherence levels and a more diverse range of risk factors. Furthermore, the limited follow-up periods characteristic of most clinical trials necessitate further exploration into the long-term effects of PrEP usage on the development of drug resistance mutation.

HIV drug resistance mutation (DRM) findings in individuals who undergo HIV seroconversion after using PrEP are often associated with the occurrence of breakthrough infections. These breakthrough infections are frequently caused by low adherence [12, 37–39]. Several studies included in our analysis, such as the iPrEx study, reported that only 17% of participants in the TDF/FTC study arm who acquired HIV had good adherence within 90 days of seroconversion [40]. Similarly, in the FEM-PrEP study, among women who seroconverted in the TDF-FTC group, the target plasma level of tenofovir (≥ 10 ng per milliliter) was identified in only 26% of women at the onset of the interaction window. These findings suggest low adherence levels among women who seroconverted to HIV during the study. This highlights the critical role of adherence in preventing breakthrough infections and the subsequent development of DRM [26, 41]. Considering these adherence challenges, Long-acting injectable cabotegravir (CAB-LA) has emerged as a potential solution. CAB-LA is a long-acting injectable form of the antiretroviral drug cabotegravir. Due to its long-acting nature, it may improve adherence by reducing the frequency of dosing compared to daily oral PrEP regimens. However, further evaluation is needed to assess the potential for DRM among individuals using CAB-LA for PrEP [42].

HIV drug resistance mutation, particularly those associated with TDF/FTC, could pose challenges following the failure of pre-exposure prophylaxis (PrEP) against HIV. Among these mutations, FTC (M184V) mutations are observed more frequently than TDF (K65R) mutations. Our study highlights that FTC (M184V) mutations are the most prevalent, constituting 33.3% of cases, within clinical trial settings of HIV oral PrEP. This finding is consistent with prior observational studies [37, 43, 44], as well as a compilation of case reports [36]. For instance, Girometti et al. reported a prevalence of 30% for the M184V mutation among individuals recently exposed to PrEP, notably higher than the 1% prevalence among those without recent PrEP exposure [37]. Similarly, a surveillance study demonstrated a notably increased risk (5.86 times higher) of M184I/V mutations among individuals with acute HIV infection (AHI) and recent PrEP usage compared to those without known usage [43]. However, the M184V mutation is not considered a severe clinical concern due to its inhibitory effect on HIV replication and subsequent reduction in viral loads among affected individuals continuing to use FTC [45]. Additionally, the presence of M184V mutations in individuals recently exposed to PrEP may be associated with the phenomenon known as the decay of PrEP-selected resistance [27]. A study has shown that the resistance developed during PrEP use tends to diminish to undetectable levels of resistance within six months after stopping the medication. This undetectable resistance persists for at least 24 months, highlighting its transient nature [27]. However, it presents challenges in resource-limited settings with limited nucleoside reverse transcriptase inhibitor (NRTI) options for constructing first-line regimens.

Our study conducted a subgroup analysis focusing on different sexual orientation populations to assess the prevalence of DRM in HIV-infected individuals who use oral pre-exposure prophylaxis (PrEP) within RCTs. The investigation revealed comparable rates of DRMs between heterosexuals and men who have sex with men (MSM) participants, with respective rates of 5.53% and 7.47%, suggesting similarity rather than disparity between the two groups. The significance of these findings lies in their insights into the risk of DRMs associated with the use of oral PrEP across different population groups. The results suggest that the risk is not significantly influenced by the sexual orientation of the user, which is a crucial consideration for healthcare providers and policymakers in scaling up the implementation of PrEP as an effective HIV preventive measure. Our findings are consistent with several recent studies investigating DRM among PrEP users. For instance, Smith et al. and Hansson et al. reported comparable rates of DRM in PrEP users, irrespective of sexual orientation [46, 47]. However, our findings diverge from those of Li et al. (2016), who reported a higher prevalence of DRM among MSM compared to heterosexuals using PrEP [48]. This discrepancy could be attributed to variations in sample size, study design, and population characteristics across different studies. Nonetheless, it underscores the importance of further research to elucidate the factors contributing to differences in drug resistance mutation prevalence among PrEP users across various demographic groups. In terms of gender-related implications, several authors have reported that women are at a higher risk of HIV acquisition and DRM compared to men [49]. However, more evidence is needed to confirm these claims within the context of pre-exposure prophylaxis.

A notable incidence of HIV drug resistance mutation (DRM) in pre-exposure prophylaxis (PrEP) was observed during the screening process for enrolment [13, 14, 30, 31, 33, 35, 40, 50]. Remarkably, our investigation revealed that the pooled prevalence of DRM during the screening process for enrolment was 4.07% across all studies included and 2.48% specifically within RCTs studies, surpassing that observed within the after-enrolment group. In contrast to several other reports [17, 51], certain instances of HIV seroconversions were classified within < 4–8 weeks in trial participants with undiagnosed acute infections. Consequently, it can be inferred that these DRM among seroconversions are not attributable to the effects of PrEP. Several factors, including delayed antibody maturation in PrEP and less sensitive diagnostic assays, are believed to be associated with undiagnosed acute HIV infections [30]. A more sensitive fifth-generation HIV antigen/antibody detection kit could be a better alternative. These include studies that use multiple magnetic bead sets coated with p24 monoclonal antibodies and epitopes specific to HIV-1 (groups M, N, and O) and HIV-2. The multiplex flow immunoassay design of the kit allows simultaneous detection, thereby reducing the window period to less than two weeks. This diagnostic assay is faster than the third-generation HIV-1 antigens/antibodies [52, 53].

Considering the low prevalence of DRMs, PrEP can be widely used according to the WHO recommendations. Consistent with these conditions, we found that PrEP did not increase the risk of developing DRM in our study (p > 0.05). This finding strengthened previous meta-analysis reports, which reported that the PrEP group had a 3.14-fold higher risk than the placebo group, but the difference was not statistically significant (p = 0.21) [17]. However, this information needs to be treated with caution as it has only been observed in the context of clinical trials. Longitudinal observations are required to understand the long-term two-way effects of DRM and PrEP.

This study has several limitations. First, the average number of included clinical trials did not examine DRM as the primary outcome, indicating limited information. Second, the information was obtained from a particular time sequence; therefore, it is necessary to continue longitudinal observations to determine the long-term impact of PrEP on DRM. Third, there is limited information regarding the DRM profiles of discordant partners, which influence high-risk exposure. Fourth, the number of included studies was limited, with some having zero arms for DRM.

## Conclusions

Owing to the increased use of PrEP, DRM has become a concern. However, our meta-analysis reveals a low prevalence of DRM among PrEP users, suggesting a relatively low risk of DRM associated with PrEP. Furthermore, we did not observe significant evidence indicating an increasing risk of DRM in PrEP users when compared with non-PrEP users. These findings underscore the importance of extending PrEP programs to high-risk HIV-negative populations and complemented by robust surveillance systems, adherence support interventions, and integration with existing HIV treatment programs. Additionally, efforts should be directed toward enhancing data sharing, collaboration, and capacity building in drug resistance mutation testing and surveillance. Besides that, it remains crucial to identify additional potential risk factors for DRM among PrEP users and to conduct longitudinal studies to elucidate these findings further.

### Supplementary Information

Supplementary material 1.

Supplementary material 2.

## Data Availability

The datasets used and/or analyzed during the current study are available from the corresponding author upon reasonable request.

## Abbreviations

ADR: Adverse drug reaction
ART: Anti-retroviral therapy
BLIND: The double-blind randomized phase
CAB-LA: Long acting injectable Cabotegravir
CI: Confidence interval
DRM: Drug resistance mutation
ES: Effect size
FDA: Food drug administration
FTC: Emtricitabine
HIV: Human immunodeficiency virus
I^2^: Heterogeneity
ICAI: Insertive condomless anal intercourse
MeSH: Medical subject headings
MSM: Men who have sex with men
NA: Not available
NGS: Next-generation sequencing
OLE: The open-label phase
OR: Odds ratio
*P* value: Probability value
PCR: Polymerase chain reaction
PrEP: Pre-exposure prophylaxis
PRISMA: Preferred reporting items for systematic reviews and meta-analyzes
RCT: Randomized-controlled trials
RT: Reverse transcriptase
RoB 2: Risk of bias 2
SE: Standard error
SNP: Single nucleotide polymorphisms
TAF: Tenofovir alafenamide
TDF: Tenofovir disoproxil fumarate
TDR: Transmitted drug resistance
TFV: Tenofovir
UDS: Ultra-deep sequencing
WHO: World Health Organization
