# Supplementary material for: Low clinical impact of HIV drug resistance mutations in oral pre-exposure prophylaxis: a systematic review and meta-analysis
Source: AIDS Res Ther. 2024 Jun 6;21:37. doi: 10.1186/s12981-024-00627-2 (PMC11155065; doi:10.1186/s12981-024-00627-2)
Supplement: Supplementary file 1 — Supplementary material 1. [file 12981_2024_627_MOESM1_ESM.docx]

**Low clinical impact of HIV drug resistance mutations in pre-exposure prophylaxis: A systematic review and meta-analysis**

**Supplementary Appendix**

Contents

[S1 Table. Search Strategies 2](#_Toc163318141)

[S2 Table. The frequency of all mutations and TDF/FTC-associated drug resistance mutations included in the meta-analysis of studies of people initiating pre-exposure prophylaxis. 3](#_Toc163318142)

[S3 Table. Sensitivity analysis (leave-one-out method) 3](#_Toc163318143)

[S1 Fig. Quality assessment results of included studies using the Risk of Bias 2 tool. (A) Detailed quality assessment summary (B) Domain-specific quality assessment graph. 4](#_Toc163318144)

[S2 Fig. Forest plot to estimate the pooled prevalence of TDF/FTC-associated drug-resistance mutation at enrolment with 95% CI (the estimate weighted based on the random effects model). ES, Effect Size equivalent to prevalence; CI, confidence interval. 5](#_Toc163318145)

[S3 Fig. Funnel plot of pooled prevalence for all mutations 5](#_Toc163318146)

[S4 Fig. Funnel plot of pooled prevalence of TDF/FTC-associated drug-resistance mutation at enrolment 6](#_Toc163318147)

[S5 Fig. Funnel plot of pooled prevalence of TDF/FTC-associated drug resistance mutation in pre-exposure prophylaxis arm after enrolment 6](#_Toc163318148)

[S6 Fig. Funnel plot of the risk of TDF/FTC-associated drug resistance mutation in pre-exposure prophylaxis arm after enrolment 7](#_Toc163318149)

# S1 Table. Search Strategies

| **Database** |  | **Keywords** |
| --- | --- | --- |
| PubMed | #1 | (((((((hiv infections[MeSH Terms]) OR (HIV[MeSH Terms])) OR (hiv[Title/Abstract])) OR (hiv-1[Title/Abstract])) OR (hiv1[Title/Abstract])) OR ("hiv infect*"[Title/Abstract])) OR ("human immunodeficiency virus"[Title/Abstract])) OR ("acquired immunodeficiency syndrome"[Title/Abstract]) |
|  | #2 | (((((((Drug Resistance[MeSH Terms]) OR ("Drug Resistan*"[Title/Abstract])) OR (Mutant[Title/Abstract])) OR (Mutation*[Title/Abstract])) OR ("pre-treatment drug resistan*"[Title/Abstract])) OR (Naïve[Title/Abstract])) OR ("transmitted drug resistan*"[Title/Abstract])) OR (TDR[Title/Abstract]) |
|  | #3 | ((("Pre-exposure prophylaxis"[Title/Abstract]) OR ("Preexposure prophylaxis"[Title/Abstract])) OR (Prophylaxis[Title/Abstract])) OR (PrEP[Title/Abstract]) |
|  | #1 AND #2 AND #3 | (((((((((hiv infections[MeSH Terms]) OR (HIV[MeSH Terms])) OR (hiv[Title/Abstract])) OR (hiv-1[Title/Abstract])) OR (hiv1[Title/Abstract])) OR ("hiv infect*"[Title/Abstract])) OR ("human immunodeficiency virus"[Title/Abstract])) OR ("acquired immunodeficiency syndrome"[Title/Abstract])) AND ((((((((Drug Resistance[MeSH Terms]) OR ("Drug Resistan*"[Title/Abstract])) OR (Mutant[Title/Abstract])) OR (Mutation*[Title/Abstract])) OR ("pre-treatment drug resistan*"[Title/Abstract])) OR (Naïve[Title/Abstract])) OR ("transmitted drug resistan*"[Title/Abstract])) OR (TDR[Title/Abstract]))) AND (((("Pre-exposure prophylaxis"[Title/Abstract]) OR ("Preexposure prophylaxis"[Title/Abstract])) OR (Prophylaxis[Title/Abstract])) OR (PrEP[Title/Abstract])) |
| Cochrane |  | ((HIV OR "Human immunodeficiency virus") AND ("Drug resistance") AND (Mutation) AND ("Pre-exposure prophylaxis" OR PrEP OR “Preexposure prophylaxis”)) AND  ((HIV OR "Human immunodeficiency virus") AND (“pre-treatment drug resistant” OR Naïve) AND ("Pre-exposure prophylaxis" OR PrEP OR “Preexposure prophylaxis”)) AND  ((HIV OR "Human immunodeficiency virus") AND (Mutation) AND ("Pre-exposure prophylaxis" OR PrEP OR “Preexposure prophylaxis”)) |
| Sage | #1 | (HIV OR "Human immunodeficiency virus") AND ("Drug resistance") AND (Mutation) AND ("Pre-exposure prophylaxis" OR PrEP OR “Preexposure prophylaxis”) |
|  | #2 | (HIV OR "Human immunodeficiency virus") AND (“pre-treatment drug resistant” OR Naïve) AND ("Pre-exposure prophylaxis" OR PrEP OR “Preexposure prophylaxis”) |
|  | #3 | (HIV OR "Human immunodeficiency virus") AND (Mutation) AND ("Pre-exposure prophylaxis" OR PrEP OR “Preexposure prophylaxis”) |
| **Total** | | |

**HIV**: Human Immunodeficiency Virus; **MeSH**: Medical Subject Heading; **PrEP**: Pre-Exposure Prophylaxis; **TDR**: Transmitted Drug Resistance

# S2 Table. The frequency of all mutations and TDF/FTC-associated drug resistance mutations included in the meta-analysis of studies of people initiating pre-exposure prophylaxis.

| **References** | **All Mutations** | **Total TDF/FTC-associated DRM** | **K65R** | **K70E** | **M184V** | **M184MV** | **M184I** | **M184IV** | **M184MI** | **M184MIV** |
| --- | --- | --- | --- | --- | --- | --- | --- | --- | --- | --- |
| Thigpen MC, et al. (TDF2) [1] | 2 | 2 | 2 | - | 1 | - | - | - | - | - |
| Liegler T, et al. (iPrex) [2, 3] | 9 | 6 | 1 | - | 1 | 3 | 2 | - | - | - |
| Grant RM, et al. (FEM) [4, 5] | 5 | 5 | - | - | 4 | - | 1 | - | - | - |
| Marrazzo JM, et al. (VOICE) [6] | 4 | 4 | - | - | 3 | - | 1 | - | - | - |
| Zhang Y, et al. (HPTN 073) [7, 8] | 4 | 1 | 1 | - | - | - | - | - | - | - |
| Sivay MV, et al. (ADAPT) [9] | 4 | 3 | 2 | - | - | - | 2 | - | - | - |
| Delaugerre C, et al. (IPERGAY) [10, 11] | 5 | 0 | - | - | - | - | - | - | - | - |
| Lehman DA, et al. (Partners) [12–15] | 7 | 7 | 1 | - | 5 | - | 3 | 7 | - | - |
| McCormack S, et al. (PROUD) [16] | 2 | 2 | - | - | - | - | 1 | 1 | - | - |
| Liu AY, et al. (DEMO) [17] | 1 | 1 | - | - | - | - | - | - | 1 | - |
| Mayer KH, et al. (DISCOVER) [18] | 4 | 4 | - | - | 2 | - | - | - | - | 2 |
| Phanuphak N, et al. (Princess) [19] | 1 | 1 | - | - | - | - | 1 | - | - | - |

**DRM:** Drug Resistance Mutation**; FTC**: Emtricitabine; **TDF:** Tenofovir Disoproxil Fumarate

# S3 Table. Sensitivity analysis (leave-one-out method)

| Omitted study | Theta θ | [95% confidence interval] | | *p*-value |
| --- | --- | --- | --- | --- |
| Thigpen MC, et al. 2012 (TDF2) | 0.951 | -0.288 | 2.189 | 0.133 |
| Liegler T, et al. 2014 (iPrex) | 1.032 | -0.217 | 2.281 | 0.105 |
| Grant RM, et al. 2015 (FEM) | 1.208 | -0.117 | 2.532 | 0.074 |
| Lehman DA, et al. 2015 (Partners) | 0.712 | -0.828 | 2.252 | 0.365 |
| Marazzo JM, et al. 2015 (VOICE) | 0.945 | -0.334 | 2.225 | 0.148 |
| Delaugerre C, et al. 2018 (IPERGAY) | 1.011 | -0.234 | 2.255 | 0.111 |
| Theta θ | 0.987 | -0.200 | 2.175 | 0.103 |

# S1 Fig. Quality assessment results of included studies using the Risk of Bias 2 tool. (A) Detailed quality assessment summary (B) Domain-specific quality assessment graph.

# S2 Fig. Forest plot to estimate the pooled prevalence of TDF/FTC-associated drug-resistance mutation at enrolment with 95% CI (the estimate weighted based on the random effects model). ES, Effect Size equivalent to prevalence; CI, confidence interval.


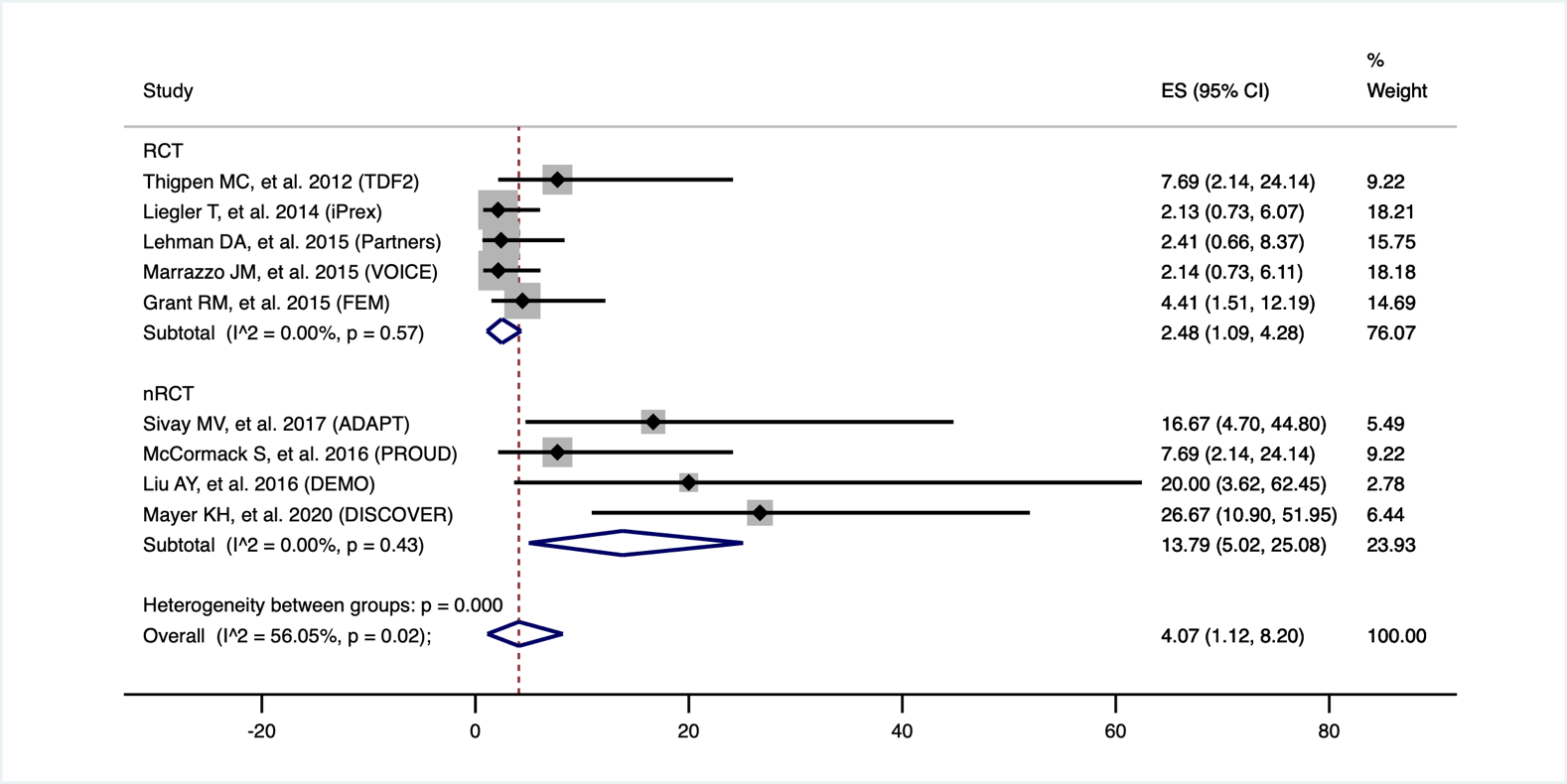


# S3 Fig. Funnel plot of pooled prevalence for all mutations

# S4 Fig. Funnel plot of pooled prevalence of TDF/FTC-associated drug-resistance mutation at enrolment

# S5 Fig. Funnel plot of pooled prevalence of TDF/FTC-associated drug resistance mutation in pre-exposure prophylaxis arm after enrolment

# S6 Fig. Funnel plot of the risk of TDF/FTC-associated drug resistance mutation in pre-exposure prophylaxis arm after enrolment

**References**

[1] Thigpen MC, Kebaabetswe PM, Paxton LA, et al. Antiretroviral Preexposure Prophylaxis for Heterosexual HIV Transmission in Botswana. *http://dx.doi.org/101056/NEJMoa1110711* 2012; 367: 423–434.

[2] Liegler T, Abdel-Mohsen M, Bentley LG, et al. HIV-1 Drug Resistance in the iPrEx Preexposure Prophylaxis Trial. *J Infect Dis* 2014; 210: 1217–1227.

[3] Grant RM, Lama JR, Anderson PL, et al. Preexposure Chemoprophylaxis for HIV Prevention in Men Who Have Sex with Men. *New England Journal of Medicine* 2010; 363: 2587–2599.

[4] Grant RM, Liegler T, Defechereux P, et al. Drug resistance and plasma viral RNA level after ineffective use of oral pre-exposure prophylaxis in women. *AIDS* 2015; 29: 331–337.

[5] Van Damme L, Corneli A, Ahmed K, et al. Preexposure Prophylaxis for HIV Infection among African Women. *http://dx.doi.org/101056/NEJMoa1202614* 2012; 367: 411–422.

[6] Marrazzo JM, Ramjee G, Richardson BA, et al. Tenofovir-Based Preexposure Prophylaxis for HIV Infection among African Women. *http://dx.doi.org/101056/NEJMoa1402269* 2015; 372: 509–518.

[7] Zhang Y, Clarke W, Marzinke MA, et al. Evaluation of a multidrug assay for monitoring adherence to a regimen for HIV preexposure prophylaxis in a clinical study, HIV prevention trials network 073. *Antimicrob Agents Chemother*; 61. Epub ahead of print 1 July 2017. DOI: 10.1128/AAC.02743-16.

[8] Wheeler DP, Fields SD, Beauchamp G, et al. Pre-exposure prophylaxis initiation and adherence among Black men who have sex with men (MSM) in three US cities: results from the HPTN 073 study. *J Int AIDS Soc*; 22. Epub ahead of print 1 February 2019. DOI: 10.1002/JIA2.25223.

[9] Sivay M V., Li M, Piwowar-Manning E, et al. Characterization of HIV seroconverters in a TDF/FTC PrEP study: HPTN 067/ADAPT. *J Acquir Immune Defic Syndr* 2017; 75: 271.

[10] Constance Delaugerre, Christophe Rodriguez, Catherine Capitante, et al. Drug resistance among patients who acquired HIV infection in a preexposure prophylaxis trial. *AIDS* 2018; 32: 2353–2361.

[11] Molina J-M, Capitant C, Spire B, et al. On-Demand Preexposure Prophylaxis in Men at High Risk for HIV-1 Infection. *New England Journal of Medicine* 2015; 373: 2237–2246.

[12] Lehman DA, Team the PPS, Baeten JM, et al. Risk of Drug Resistance Among Persons Acquiring HIV Within a Randomized Clinical Trial of Single- or Dual-Agent Preexposure Prophylaxis. *J Infect Dis* 2015; 211: 1211–1218.

[13] Heffron R, Ngure K, Odoyo J, et al. Pre-exposure prophylaxis for HIV-negative persons with partners living with HIV: uptake, use, and effectiveness in an open-label demonstration project in East Africa. *Gates Open Res*; 1. Epub ahead of print 2018. DOI: 10.12688/GATESOPENRES.12752.2.

[14] Baeten JM, Donnell D, Ndase P, et al. Antiretroviral Prophylaxis for HIV Prevention in Heterosexual Men and Women. *http://dx.doi.org/101056/NEJMoa1108524* 2012; 367: 399–410.

[15] Baeten JM, Donnell D, Mugo NR, et al. Single-agent tenofovir versus combination emtricitabine plus tenofovir for pre-exposure prophylaxis for HIV-1 acquisition: an update of data from a randomised, double-blind, phase 3 trial. *Lancet Infect Dis* 2014; 14: 1055–1064.

[16] McCormack S, Dunn DT, Desai M, et al. Pre-exposure prophylaxis to prevent the acquisition of HIV-1 infection (PROUD): effectiveness results from the pilot phase of a pragmatic open-label randomised trial. *Lancet* 2016; 387: 53–60.

[17] Liu AY, Cohen SE, Vittinghoff E, et al. HIV Pre-Exposure Prophylaxis Integrated with Municipal and Community Based Sexual Health Services. *JAMA Intern Med* 2016; 176: 75.

[18] Mayer KH, Molina JM, Thompson MA, et al. Emtricitabine and tenofovir alafenamide vs emtricitabine and tenofovir disoproxil fumarate for HIV pre-exposure prophylaxis (DISCOVER): primary results from a randomised, double-blind, multicentre, active-controlled, phase 3, non-inferiority trial. *Lancet* 2020; 396: 239.

[19] Phanuphak N, Sungsing T, Jantarapakde J, et al. Princess PrEP program: the first key population-led model to deliver pre-exposure prophylaxis to key populations by key populations in Thailand. *Sex Health* 2018; 15: 542–555.
